# Supplementary material for: Are carbon emissions trading and green financial instruments synergistic? -Comprehensive quantitative research based on content analysis
Source: PLoS One. 2024 Mar 7;19(3):e0298601. doi: 10.1371/journal.pone.0298601 (PMC10919601; doi:10.1371/journal.pone.0298601)
Supplement: S1 Data — (ZIP) [file pone.0298601.s001.zip › Data/地级市Z_F工作报告.zip!/║┌┴·╜¡/╦τ╗»╩╨/└┤╫╘╣1⁄2╓┌║┼í╛┬φ┐╦╩2╛▌═°í┐_2017.docx]

各位代表:

现在，我代表市人民政府，向大会报告工作，请予审议，并请各位市政协委员提出意见。

一、过去五年工作回顾

市三届人大一次会议以来，在省委、省政府和市委的正确领导下，我们认真贯彻落实习近平总书记系列重要讲话精神,特别是对龙江两次重要讲话精神，开拓创新，攻坚克难，胜利完成“十二五”规划，“十三五”规划开局良好，为实现全面建成小康社会宏伟目标奠定坚实基础。

综合实力持续增强。2016年，实现地区生产总值1371亿元，是2011年的1.5倍，年均增长8.7%；固定资产投资766.6亿元，是2011年的1.8倍，年均增长12.9%；规模以上工业增加值200亿元，是2011年的1.5倍，年均增长8%；社会消费品零售总额567.6亿元，是2011年的1.9倍，年均增长14.1%；公共财政预算收入55.8亿元，是2011年的1.3倍，年均增长5.8%。主要发展指标保持较快增长，居于全省领先位次。五年开工建设产业项目2741个，完成投资1721亿元。绥化经济技术开发区晋升为国家级开发区，各县（市）开发区均进入省级开发区或省级工业示范基地行列。被确定为全国粮食生产先进市和国家级现代农业示范区。肇东、安达综合实力位列全省前三，跻身全国百强县。

产业结构进一步优化升级。农业现代化步伐加快。粮食生产实现“十三连丰”，粮食综合产能稳定在300亿斤以上，10个县（市、区）全部进入全国产粮大县行列。绿色食品产业快速发展，认证面积1180万亩，占播种面积的41%。产业化水平明显提升，加工能力1500万吨，加工转化率65.7%。畜牧业快速发展，增加值225亿元，是2011年的1.5倍，畜牧业增加值占农业比重提高到45%。基础设施进一步完善，水利建设投资73.8亿元，是前五年的3.3倍。工业实力加速提升。规模以上企业由2011年的167户发展到376户，产值、利税增长2.3倍和1.2倍。绿色食品和农产品加工产业规模全省第一，医药产业规模全省第二。高新技术产业增加值56.7亿元，增长10.6%。非公经济增加值775亿元，占地区生产总值的57%。第三产业增加值459.3亿元，是2011年的1.5倍，现代物流、电子商务、金融信息等现代服务业快速发展。

城乡建设和基础设施更加完善。常住人口城镇化率达到45.4%，比2011年提高6.4个百分点。现代交通较快发展，北大荒通用航空肇东机场投入运营，伊绥高速、绥北高速建成通车，绥大高速、哈黑公路兰明青段开工建设，绥化机场、哈绥城铁启动前期工作，升级改造国省干道463公里，新建改建农村公路4269公里，初步形成便捷快速的交通网络。城市功能不断完善，房地产开发面积1921万平方米，完成投资560亿元。市政基础设施完成投资116.9亿元，是前五年的1.7倍。村镇建设步伐加快，完成投资324亿元，是前五年的2倍。青冈、安达、绥棱列入国家新型城镇化综合试点，26个小城镇进入全国重点镇。累计投入3.1亿元，对470个村进行环境集中连片整治。绿化覆盖率达到31.2%。

改革创新取得新成果。现代农业综合配套改革深入推进，土地确权登记完成实测，新型经营主体达到4.9万个，是2011年的3倍。土地规模经营1810万亩，占耕地面积63%。放管服改革不断深化，全市精简审批事项2万多项，非行政审批事项全部清零。权力清单、责任清单向全社会公布。县级公立医院综合改革全面推开。行政机关公务用车制度改革基本完成。机关事业单位养老保险制度改革稳步推进。

民生和社会事业明显改善。城乡常住居民人均可支配收入分别实现21903元和11903元，是2011年的2倍和1.5倍。民生支出占财政支出比重达到74.6%。累计新建保障性安居工程822万平方米，11万户城市居民、23.4万户农村居民喜迁新居。新建改造学校幼儿园430所，义务教育学校标准化率提高到74.7%。安达顺利通过农村义务教育均衡发展国家验收。新改扩建县级以上医疗机构26家、乡镇卫生院96所、村卫生室585个。新增养老机构78个、床位1.3万张。城乡低保应保尽保。城镇登记失业率始终低于全省平均水平，各项社会保险待遇逐年提高。扶贫开发取得重大成果，313个村、35.7万贫困人口实现脱贫。

政府自身建设不断加强。深入开展党的群众路线教育实践活动、“三严三实”专题教育、“两学一做”学习教育。建立双周学习制度，健全完善政府法律顾问和专家课题组制度。改善经济发展环境，26个部门179项行政许可实行告知承诺制，选派299名后备干部任“驻企秘书”。认真履行“一岗双责”，严格落实中央八项规定精神，坚决反对“四风”，强化行政监察和审计监督，政府系统党风廉政建设取得扎实成效。

刚刚过去的2016年，全市上下学讲话、谋振兴、闯新路，积极做好“三篇大文章”，重点工作取得新成效、新突破。一是产业项目建设保持强劲态势。签约引进产业项目801个，开复工761个，大庄园全产业链项目、象屿粮食仓储加工、金达亚麻、胜记仓北方商谷等一批大项目开工建设。二是农业发展方式转变成效明显。调减玉米面积13.7%，落实“三减”示范面积570万亩。加大“寒地黑土”等品牌建设力度，加快由种得好向销得好转变，把畜牧产业上升到战略性支柱产业来抓，农民创业取得较大突破。三是基础设施建设取得重大进展。采取PPP模式投资105亿元的绥大高速和投资3亿元的绥巴路开工建设。投资38.3亿元的阁山水库正式开工，十六道岗水库列入国家水利改革发展“十三五”规划。投资30亿元的大唐热电联产项目进展顺利。四是新经济新业态新商业模式迅速兴起。全市电子商务企业、个体经营者突破1万家，建设网站、网馆及电商平台46个，开设营销店铺7000多个，明水、庆安、安达、兰西被评为全国电子商务进农村综合示范县。引进万达集团建设大型商贸综合体。组建寒地黑土品牌农业、城市发展、“双创”三支产业发展基金。“新三板”挂牌企业达到7户。五是民生保障水平进一步提高。加大老旧小区、社区、物业、供水、供热等改造力度，实施绿化美化亮化工程，提高城乡低保、社会救助补贴标准，加强城乡环境综合整治，认真解决群众关心的热点难点问题。

同时，信访稳定、公共节能、农业开发、民族宗教、财税金融、规划国土、外事侨务、食药监督、市场监管、国防动员、妇女儿童、统计监测、助残慈善、防灾减灾、市志档案、气象人防、老龄双拥等各项工作都取得了新进步。

各位代表！回首过去五年，发展令人欣喜。这是省委、省政府和市委正确领导的结果，是全市人民拼搏奋斗的结果，是社会各界鼎力支持的结果。在此，我代表市人民政府，向全市人民，向各民主党派、工商联、人民团体和各界人士，向中省直单位、驻军部队、武警官兵，表示衷心感谢，并致以崇高敬意！

五年的实践探索，让我们更加深刻地体会到，政府工作必须坚决贯彻落实党中央、国务院和省委、省政府的决策部署，紧紧依靠市委的坚强领导，紧紧依靠全市人民的广泛支持；必须抓牢产业项目不放松，思路不变、重点不移、力度不减；必须立足资源和优势做文章，扬长避短、扬长克短、扬长补短，在“农头工尾”“粮头食尾”“油头化尾”上聚焦发力；必须坚持以人民为中心，办实事、解难题，不断增进人民群众福祉；必须苦干实干，滚石上山，爬坡过坎。这些，一定要在今后的政府工作中紧紧把握、牢牢坚持，不断发扬光大。

面对新常态、新理念、新要求，我们清醒地认识到还存在很大差距不足。优势发挥不够充分，产业规模小，没有形成聚集效应。我市优质农牧资源丰富，但开发利用不足、水平低，跟总书记“农头工尾”“粮头食尾”的要求差距很大，资源优势没有转换成产品优势和产业优势。半数以上农产品处于粗加工，成为别人的原料车间，玉米等还形成了大量的库存。绥化是距哈尔滨最近的地级市，处在全省发展要素最富集的黄金产业带上，但融入都市圈、连接产业带还不够，区位优势没有转化为发展优势。资源潜力尚未有效释放，产业链、价值链短。我市绿色食品面积全国地级市总量第一，但我们捧着寒地黑土这个金饭碗，却还没有过上富裕日子。绿色食品产业普遍处于产业链、价值链、供应链中低端，头大尾小、量大链短、种强销弱，产业结构趋同、科技含量低、附加值低问题突出。经济结构不尽合理，结构调整优化升级刻不容缓。突出表现为工业不强、城市经济不够繁荣。市本级经济规模小、牵动力不强，产城没有有效融合。第一产业比重高，传统玉米、水稻等农产品大路货多，市场需求空间大的特色绿色农产品没有形成规模，如何把“多”的去掉，把“少”的做强，还没有完全破题。工业比重低，够块头的国内外大企业来的还不多，缺少支柱骨干企业；产品附加值低，企业盈利水平普遍不高，有的靠政府补贴才能维持开工生产，对财政贡献和农民增收作用不大。第三产业中传统服务业、生活服务业比重较高，现代服务业特别是生产性服务业滞后。新经济发展缓慢，新动能不强，农区思维、传统意识较重，活力不足。经济增长主要还依靠传统模式和增长方式驱动，新领域、新产品、新市场开发不足，新技术、新业态、新商业模式发展滞后，互联网+、大数据、云计算等与实体经济联接不紧密，新体制、新制度、新机制、新思路、新政策还没有充分激发释放增长动力和活力。基础设施和民生短板较多，城市风格不鲜明。中心城市建设欠账较多，规划滞后、建设拖后、管理落后的问题还不同程度存在；基础建设、公益设施大多依靠上级支持；优质教育、医疗、社会福利资源供给不足，特别是市本级缺少大型会展中心、文体中心、图书馆等公益设施；城市特色不明显，缺少记忆和回味；一些事关百姓切身利益问题解决的还不够好，人民群众的合理诉求解决的还不到位，一些社会矛盾隐患较大。行政运行效率不高，政府系统工作作风急需进一步转变。市县乡三级政府到农村和企业的“最后一公里”问题还没有解决好，一些政府部门相互掣肘、关键环节堵塞而导致整个链条运转不畅；一些政府工作人员作风不实，浮在表面，热衷于锦上添花、不愿意雪中送炭，热衷于交人做好事，不愿意触及矛盾主动担当，热衷于形式主义，不愿意脚踏实地埋头苦干；一些政府工作人员缺乏“工匠精神”，专业水平不高，能力不足，老办法不管用、新办法不会用的问题比较普遍；一些政府工作人员不作为、慢作为、乱作为，严重影响了工作效率和发展环境。

我们要客观面对这些问题，客观面对城镇化水平全省最低，贫困人口全省最多，还处于农区工业化、城镇化、现代化初中期的现实，想问题干事情都应保持更加清醒的头脑，以更加科学的谋划、更加务实的作风、更加有效的措施，一步一个脚印、踏踏实实往前走。

二、未来五年目标任务

各位代表！未来五年，既是我市经济社会发展重要的战略机遇期，也是经济结构转型升级的攻坚期，更是实现全面振兴发展的关键期。有挑战、有机遇，有困难、更有希望。党中央国务院稳中求进的总基调使我们有了基本遵循，也为我们产业转型升级，提高质量效益，赢得了时间和空间；供给侧结构性改革、农村集体产权制度改革以及其他重点领域改革，有利于土地、资本等要素充分流动，配置到回报率较低但长期收益稳定的农业领域，聚集到像绥化这样的农业地区，更有利于我们放手发展质量效益农业，推动绥化农业真正大起来、强起来；我们没有更多的国有资产可以继承，但同时也没有更多卸不掉的包袱和体制束缚，完全可以轻装上阵，快马加鞭，实现动能转换、弯道超车；哈长城市群规划、东北振兴战略、脱贫攻坚等政策含金量大，指向精准，个个都与绥化发展密切关联，有利于推动经济交流合作，承接产业转移，实现贫困落后地区基础设施翻天覆地的变化，为我们带来实实在在政策红利；绿水青山、冰天雪地和不可复制的寒地黑土资源，可以生产出世界公认的优质绿色安全食品，这与国内外巨大的市场需求高度契合，与重点扶持发展实体经济的要求高度契合，绥化的优势更加凸显，寒地黑土成为绥化最响亮的名片，必将吸引更多的海内外、省内外大企业、大财团的目光，刺激更大投资需求；勤劳纯朴、包容善良的绥化人民所传承的文明和谐、大爱无疆的美德，成为绥化发展最可贵的人文环境和不竭的动力源泉。党政同心、上下同力，人心思进、人心思变的政治氛围成为发展最坚强的政治保障！这样的历史机遇千载难逢，坚决不能错过！市第四次党代会明确提出了今后五年全市的发展思路、目标任务，号召全市上下凝心聚力、砥砺前行、决战决胜，奋力谱写绥化全面振兴发展新篇章。政府工作要全面、系统、准确贯彻落实市第四次党代会精神。深入贯彻党的十八大和十八届三中、四中、五中、六中全会精神，以习近平总书记系列重要讲话精神特别是对龙江两次重要讲话精神为指针，紧紧围绕“五位一体”总体布局和“四个全面”战略布局，以新发展理念为引领，适应经济发展新常态，坚持稳中求进总基调，深入贯彻落实省委“五大规划”、“十大重点产业”、“龙江丝路带”建设和坚决打赢脱贫攻坚战等一系列重大决策部署，全力打造寒地黑土之都、绿色产业之城、田园养生之地，走好创新、协调、绿色、开放、共享五条发展新路子，使经济更加发展、政治更加清明、文化更加繁荣、社会更加和谐、生态更加优良，夺取全面建成小康社会决战决胜，在绥化全面振兴发展的新征程上创造出更加辉煌的业绩。

主要发展目标是：地区生产总值年均增长7.5%；规模以上工业增加值年均增长8%；固定资产投资和社会消费品零售总额年均增长10%；公共财政预算收入年均增长8%；城乡常住居民人均可支配收入年均增长8%。“十三五”规划全面完成，贫困人口全部脱贫，与全省、全国一道全面建成小康社会。

坚持创新驱动，激发内生动力，转换发展动能，加快转型升级，在创新发展上迈出新步伐。着力提高农业质量效益和竞争力。以农业供给侧结构性改革为主线，依靠科技创新、机制创新、模式创新，推进一二三产业融合，围绕粮食生产功能区、重要农产品生产保护区、特色农产品优势区建设，放大寒地黑土优势，发展品牌农业，把绿色食品产业作为第一大产业，建设亚洲最大玉米精深加工中心，全国知名“好吃米饭”生产基地，中国绿色蔬菜“北菜南运”核心区，打造绿色粮仓、绿色菜园、绿色厨房，扩大寒地黑土之都影响力，在争当全国农业现代化建设排头兵上走在全省前列。在“五个要发展”上聚焦发力。持续推进产业项目建设，构建1+4+N产业格局。抓好绿色食品、农产品加工和精细化工等重点产业扩张规模、延长链条、补齐短板，引进战略投资者，做好“三篇大文章”，在“农头工尾”“粮头食尾”“油头化尾”上重点突破，打造1个千亿级产业、3个百亿级园区、50个十亿级企业群体，建设一批优势特色产业加工板块，推进绿色产业之城建设，打造龙江腹地富有生机活力的成长带。培育壮大新经济。发展新技术、新业态、新商业模式，推进现代物流、金融、信息等生产性服务业加快发展，推进健康养老旅游融合，发展大健康产业，建设服务龙江、服务全国的田园养生之地。到2021年，三产结构比达到36:26:38。畜牧业增加值占农业比重达到50%以上。农产品精深加工率达到50%以上。经济结构发生深刻变化，发展活力和动能持续增强，经济发展迈上新台阶。

坚持系统思维，城乡区域统筹，经济社会同步，人与自然和谐，在协调发展上取得新成效。发展城市经济，做美做强做大中心城市。以开发区为主体加快转型升级，建设主导产业核心区；以东富工业园区为基础建设现代物流城；以中心城区为平台建设现代商贸中心区；以西部新区为重点发展现代服务业功能区；以宝山镇为载体打造养老养生大健康产业聚集区和现代农业服务示范基地，构建多元支撑的产业体系。完善城市整体功能。积极推进行政区划调整，加快城市扩容，拉开城市框架，提升城市品位。构建立体开放交通网络，打造“半小时核心圈”、“一小时经济圈”。推进西部新区、东部新城协调发展，加快开发区产城融合，构建海绵城市和城市水系，建设高水平现代生态园林城市。提高公共服务水平，推进高校、医院、科研机构设立分支机构或合作发展，增强服务能力。建设成为具有较大影响力和知名度的哈尔滨都市圈副中心城市、龙江中部区域中心城市。发展县域经济，做特做优做强县市区。推进产城融合、错位发展，加快优势要素向优势资源聚集，建设专业园区，打造特色加工板块，加快人口聚集和生产生活功能配套，提升整体功能，构建竞相发展新格局。发展镇村经济，做精做专做好特色镇村。加快以产业为基础的特色小镇建设，提升美丽乡村建设层次，大力发展民宿经济，把绥化全域建设成为知名的体验式旅游、候鸟式养老、田园式养生基地。到2021年，全市常住人口城镇化率达到50%。实现市县乡协调发展，产城人有机融合，城乡环境更加宜居宜业。

坚持永续发展，壮大循环经济，推进节能减排，建设生态家园，在绿色发展上实现新突破。坚持绿色生产，发展生态农业，加强黑土地保护，扩大“三减”面积，探索有序休耕轮作模式，抓好呼兰河流域综合开发，推进全域生态工程建设。坚持绿色制造，用高新技术改造传统产业，降低能源消耗和污染排放，加强新能源汽车推广应用，发展循环经济，坚决不上环保不达标的项目。坚持绿色消费，转变生产生活方式，实施生态修复、城市修补。实行最严格的环境保护制度，推进大气、水、土壤污染防治行动。到2021年，全市万元生产总值能耗年均下降3.2%，大气污染排放物下降10%，森林覆盖率达到13%，秸秆综合利用率达到90%。空气质量良好天数达到320天以上。绿水青山、冰天雪地、寒地黑土的优势潜能不断放大，生态示范区建设取得明显成效。

坚持合作共赢，推进内引外联，借势借力借智，融入服务支撑，在开放发展上拓展新空间。主动融入“龙江丝路带”建设，提高基础设施互联互通能力，积极参与哈大齐（满）和哈绥北黑产业聚集带建设，主动对接全省陆海联运大通道，提高通关便利化水平。加强对俄、日、韩、澳等国家经贸交流和技术合作。培育壮大外向型企业群体，重点打造特色农产品、畜产品、精细化工出口产业基地和跨境产业园区。推进区域合作，加强与广东市域间对口合作，拓展合作领域，提升合作层次。推进地方国有、民营企业与央企竞争性产业链合作，深化与中粮集团、大唐集团等央企国企合作。加强与哈尔滨、大庆合作，加快产业共建、市场共享、人才共用。今后五年，全市外贸进出口总额和实际利用外资年均增长7%以上，有出口实绩企业增长1倍以上。加快融入产业带、都市圈经济，在更大范围内优化资源、配置要素，经济发展外向化水平进一步提高。

坚持以人民为中心，维护公平正义，回应群众关切，办实事解难题，在共享发展上得到新提升。坚决打好打赢脱贫攻坚战，6个贫困县、200个贫困村、14.7万贫困人口全部告别贫困。深入实施惠民利民行动，认真解决就业、上学、看病、住房、出行、食品安全等关系人民群众切实利益的民生问题，提高社会事业发展水平。基本消灭城市棚户区、农村泥草房。多渠道增加城乡居民收入，提高中低收入群体生活水平，提高城乡低保补贴标准，稳步提高职工平均工资水平。民生投入占财政支出比重达到78%。高等教育在学人数占学龄人口总数达到45%以上，每千人口医院床位数增长25%以上。在群众最迫切的需求上下功夫，在群众最忧虑的问题上出实招，让百姓更幸福，让绥化更和谐。

各位代表！五年的目标任务，描绘了绥化未来发展的美好愿景，体现了全市人民的共同期待。展望未来，信心前所未有！我们坚信，经过五年不懈努力，绥化经济发展一定会有一个大的进步，人民生活水平一定会有一个大的提高，绥化明天一定会更加美好！

三、2017年重点工作

2017年，是新一届政府的开局年，是转型升级的关键年，更是弯道超车的起步年。要在市委坚强领导下，合力攻坚、奋力拼搏，全面开启振兴发展新征程。

主要发展目标是：地区生产总值增长6.5%-7%；固定资产投资增长8%；规模以上工业增加值增长7%；社会消费品零售总额增长10%；外贸进出口总额增长7%；公共财政预算收入增长5%；城乡常住居民人均可支配收入增长6%。

重点做好以下八个方面工作：

（一）加大招商引资力度，在产业项目建设上聚焦发力。坚持精准招商。继续把产业项目建设作为一号工程强力推进，掀起新一轮招商引资项目建设热潮。加强与广东省对口合作城市的产业转移、项目投资合作。深化与哈尔滨、大庆的融合发展，推进产业吃配衔接。跟踪落实央企对接会、知名民企对接会洽谈成果。按照产业拉动、财政创税、增加就业、环境影响等因素，制定完善招商引资标准，依据标准招商选商。建立项目推进责任机制，亿元以上项目县（市、区）主要领导牵头推进。全年引进项目数、新开工项目数和投资额都要力争比上年提高15%以上。把绿色食品产业作为全市主攻方向。科学制定五年产业发展规划，在终端产品开发、品牌打造、细分市场营销上重点突破。加大玉米精深加工力度，重点引进2户大型加工企业，玉米加工能力达到500万吨，产品向酶制剂、生物医药、淀粉基塑料等方向拓展，向有机酸、乳酸、聚乳酸等终端延伸。积极引进扩大小麦粉加工能力。利用玉米价格回归市场时机，大力发展生猪精深加工项目，带动畜牧经济加快发展。提升产业园区功能。加大园区建设力度，完善园区铁路专用线、输变电和集中供热等基础设施配套，加快金融、法律、咨询等配套中介服务机构进驻。提高肇东绿色食品产业园、安达高新化工新材料产业园等园区专业化水平。加强重点企业运行服务，帮助企业降低成本，推动“千户科技型企业三年行动计划”。支持绥化经济技术开发区做大做强。强化主导产业牵动力，打造以中粮、昊天、维维等为龙头的绿色食品加工集群和以东富工业园区为重点的现代物流城。加大招商引资和老企业盘活力度，引进6个以上绿色食品精深加工项目，下大力气提升开发区综合实力。加快公共服务、投融资和创新平台建设。推进开发区向综合性园区转型，完善生活服务配套功能，与城市建设、土地利用规划调整、交通路网改造统筹安排，推进产城融合。强化领导力量，进一步理顺完善管理体制、财税体制，赋予更多的行政审批权和决策自主权。使开发区成为市本级发展的增长极、全市经济发展的新引擎、体制机制创新先行区。大力发展民营经济。制定出台更有力、更开放、更宽松的支持政策，把民营经济上升到市域经济的主体地位。按照有利于民营经济发展的标准，倒逼政府职能转变、放管服改革。鼓励支持民营经济“个转企”、“小升规”，支持与地方国有企业兼并重组、嫁接改造，发展混合所有制经济。克服歧视思想，严查勒卡行为，营造宽松环境。

（二）深化农业供给侧结构性改革，在提升农业综合效益和竞争力上聚焦发力。提高农产品供给质量，增加绿色优质农产品供给。积极与销区市场对接，调整优化种植结构。扩大加工型专用玉米和鲜食玉米种植，调减传统玉米面积100万亩，加工型专用玉米增加到780万亩，鲜食玉米超过20万亩，建成全国最大的鲜食玉米生产基地。发展绿色无公害蔬菜250万亩，推进“北菜南运”核心区建设，打造“北方寿光”。全面推广绿色标准化生产规程，落实“三减”面积700万亩。160个互联网+高标准基地全部实现全程可追溯，绿色食品种植面积达到1800万亩，有机食品面积增加2万亩。拓展营销渠道。加大农产品B2B、O2O线上交易推广力度，农产品线上交易额增长20%以上。推进会展营销，积极参加中俄博览会、绿博会等知名展会，举办绥化寒地黑土优质农产品订货会，筹建中国北方（绥化）玉米交易中心和（海伦）大豆交易中心，扩大绥化绿色农产品知名度。建立农业信息服务平台，指导农民种什么、怎么种、怎么销。鼓励支持发展“庭院经济+”，继续推进“我在绥化有亩田”、“我在绥化有菜园”订制、众筹活动。加强农业基础设施建设。加快阁山水库建设，做好十六道岗水库前期工作。加强高标准农田建设，新增生态良好、集中连片、设施完善的高标准农田60万亩。推进节水增粮行动。新增农机合作社12个。新建水稻育秧大棚6600栋，新增棚室面积2万亩。发展生态林业、产业林业、民生林业、扶贫林业，林业产值增长7.5%。完成造林绿化面积13.8万亩，森林覆盖率提高到12.6%。加快畜牧业战略性支柱产业发展。发展以“两牛一猪一禽”为重点的草食畜牧业和耗粮型畜牧业。新建规模养殖场120个，改扩建畜牧科技示范园区20个，先进科技推广面达到90%以上，规模化、规范化养殖比重提高3个百分点。新上规模生猪养殖企业5户以上，新增生猪饲养量80万头以上，积极引进大型加工企业，提高精深加工水平。新增肉牛饲养量20万头，奶牛饲养量2.5万头。进一步扩大饲草饲料面积。畜牧业增加值增长4.5%。加强职业农民培育。举办农业创新、农民创业讲习班，传授农民增收致富经验做法。出台支持农民创业意见，支持农民创业园、农民创业基地建设，创业农民新增5万人，培育农民创业领军人物200名。转移劳动力130万人，劳务收入185亿元。

（三）加快发展新经济新业态，在培育新的经济增长点上聚焦发力。实施创新驱动。大力推进“大众创业、万众创新”，鼓励支持企业建立研发机构，积极创建玉米研发中心。与绥化学院、省农科院合作，建设科技企业孵化器和“双创”园区，推进“创客大学”项目建设。搭建科研平台，力争实现省级领军人才零的突破。建立1处博士后流动工作站、10个技能大师工作室。重点引进一批急需的规划、金融、策划、管理等方面专业人才。激发方方面面创新创业积极性，广泛吸引汇聚各地人才，共同推动绥化发展。建设绿色物产通道。推进安达胜记仓北方商谷、兰西隆盛物流园等大型物流商贸平台建设，打造区域展示、交易、集散中心。加快象屿集团、京粮集团、中国供销集团大型粮食物流项目建设，积极引进第三方专业物流，依托国家粮食统一竞价交易平台，盘活现有铁路专用线、仓房等资源，在海伦、肇东、北林、庆安等地打造国家级粮食物流中心。实施“互联网+”行动计划，加快发展电商产业，推进与阿里巴巴、京东等电商集团深度合作，建立特色物产分销平台。以销区商业资本为龙头，建设寒地黑土产业联盟示范体系。推动健康养老旅游产业融合发展。重点打造肇东沿江、兰西十八湾、庆安柳河、绥棱四海店等旅游带，积极发展农业主题公园、休闲农庄、农家乐聚集区、休闲农业特色镇。推进绥棱黑陶、海伦剪纸、兰西挂钱等文化产品，以及明水小杂粮、青冈粘豆包、榆林镇筋饼等特色美食与旅游有机融合，让更多的外地人到绥化赏自然风光美景、品生态绿色美食、享幸福美好时光。积极融入“龙江丝路带”。支持兰西天纤纺、肇东成福等企业借助“哈欧”、“哈俄”班列，扩大出口规模。培育壮大外向型优势企业，支持双汇北大荒、赛美葵花、海伦中际等重点企业发展。建设玉米精深加工、肉制品、绿色食品、化工产品进出口基地。加强与哈尔滨保税区及大连等口岸城市合作，提高通关便利化水平。拓宽融资渠道。完善融资平台、担保平台建设，创新金融产品。建立企业上市孵化基地，支持重点企业登陆资本市场，力争实现“新三板”挂牌企业县域全覆盖。做好PPP项目储备工作，吸引社会资本投资。推进品牌农业、城市发展、“双创”等产业基金在年内启动运作。谋划绿色金融、绿色债券、互联网金融等融资模式，积极引进金融租赁公司、信托公司等金融机构。加快推进农村金融创新，搭建“三农”及中小微企业融资平台。每个县（市、区）新建1家以上新型农村金融组织。推进寒地黑土品牌建设。加强寒地黑土品牌研究开发，推进标准制定和使用规范，打造全市集体名片。加大“寒地黑土+北京产品”、“寒地黑土+县域产品”推介力度。加强对庆禾香、龙蛙等全国知名品牌和明水黑豆、庆安大米、肇东小米、海伦大豆等地理标识保护产品的宣传推介，让绥化地方特产在全国叫响。

（四）统筹抓好城乡建设，在新型城镇化上聚焦发力。提高中心城市建设水平。加强与哈尔滨、大庆基础设施互联互通，绥大高速全线开工，哈绥城铁完成前期工作，绥化机场有实质性进展，绥巴路10月份通车。做大做强市城投公司，增强融资建设管理功能。推进西部新区建设，规划完善西部路网，启动5个单元城市控规编制和城市设计。推进东部新城建设，加快示范性重点工程进度。推进老城区功能升级，打通黄河路、科研路，改造新兴街、花园街，配套建设祥和街、绥望路。启动城市水系规划编制，推进海绵城市、饮用水源地、地下综合管廊建设。加快城南河综合治理工程。大唐热电联产项目建成并投入使用。持续推进环境综合整治，实施城市绿化亮化美化工程，提高城市精细化管理水平。增强县城功能。编制肇东、兰西融入哈尔滨，安达融入大庆发展规划和庆安、望奎建设中心城市联动区发展规划。开工建设国省干道及农村公路改造升级项目，力争哈黑公路兰明青段升级改造10月份通车。加强市政设施建设，投资4亿元，新建改建扩建城区道路35公里、面积34万平方米，铺设排水管线18公里，改造维修泵站6座。新增绿地60公顷，新建改造公园及广场5个。提高“三供三治”保障水平，投资5.2亿元，新建和结转项目17个。肇东、海伦、兰西垃圾处理项目按要求时限建成投入使用。建设特色镇村。提升小城镇吸纳力和承载力，投资5亿元，推进基础设施、公共服务设施和环境建设。集中打造肇东五站、海伦海北、兰西榆林、庆安柳河等10个工业重镇、商贸大镇、特色强镇、旅游名镇。推进美丽乡村建设，开展改善农村人居环境示范村创建活动，“五乱”现象基本杜绝，“六改”工程取得明显成效。环境卫生管理延伸到村屯，提高城乡环境综合整治水平。加强生态环境建设。珍惜和保护绥化得天独厚的生态环境，珍视和维护在市场上取得的地位和口碑，严厉打击破坏行为，让绥化的生态环境变得更好！加强大气污染防治，10蒸吨以下燃煤小锅炉、黄标车全部淘汰。严格规范露天烧烤，依法查处肆意焚烧垃圾和废弃物的违法行为。全域禁烧秸秆。加强水污染防治，推进县以上集中式饮用水水源地规范化建设。完成肇兰新河年度重点建设任务，实现省级以上工业园区污水全部集中处理。实施新一轮退耕还林还草还湿工程。全面完成中央、省环境保护督查交办的整改事项。

（五）深化重点领域改革，在激发释放发展活力上聚焦发力。推进两大平原综合配套改革。完成土地确权颁证工作，加快土地流转平台、抵押贷款平台和产权交易平台建设。积极推进村集体组织成员身份和承包关系确定工作。培育新型经营主体，创建示范社50个，每个乡镇培育1个以村为基本单元复合型经营主体。积极探索资源变资产、资金变股金、农民变股民。推进新型经营主体与国有粮库、供销社等传统经营主体融合发展。探索发展农村集体经济有效实现形式，推进清产核资、股权量化，村集体收入增长5%以上。推进放管服改革。动态调整权力清单、责任清单、中介服务清单，全面推行告知承诺制，推动中介机构改制脱钩。推行网上办公，实现省市县三级联网审批。加快市人民办事中心、不动产登记中心、公共资源交易中心和企业投诉中心建设。医疗、教育、公共网络、公共交通、城市基础设施等公共资源,全部采取市场化招标等公开方式进行配置。推进医药卫生体制改革。全面推开城市公立医院综合改革，实现医药分开。整合城乡居民基本医疗保险，完成城乡居民医保政策统一工作。推进民生价格改革。实施阶梯水价、工商业水价改革，调整市区物业收费标准，实行非居民用水累进加价制度和煤炭供热价格联动机制。推进城市管理执法体制改革。明晰城市管理职责，整合执法力量，改进执法方式，推进综合执法。同时，抓好农垦、铁路社会职能分离承接工作。对一些影响发展的体制机制矛盾都要坚决用改革的办法加以解决。

（六）坚持精准扶贫，在确保稳定脱贫上聚焦发力。加大产业扶贫力度。重点发展畜牧、水产、蔬菜、杂粮、食用菌、中草药、林下经济等产业，实现户户有脱贫项目。引导享受政府扶持政策的企业承担社会责任，把产业基地建在贫困村，对接到贫困户，增加贫困人口在生产、销售环节的收益。新上扶贫开发特色产业项目3000个，带动5万以上贫困人口创业就业。筑牢保障底线。加大投入力度，实现助学补助补贴、医疗保险、大病救助全覆盖。确保退出的贫困户人均纯收入持续稳定超过国定省定贫困线，预出列的贫困村实现“三有五通”。强化扶贫力量。健全完善稳定脱贫长效机制、工作考核机制和责任追究机制。坚持脱贫攻坚组织领导双组长制不变，行业部门包联一包到底不变，驻村工作队和第一书记包扶对象不脱贫不脱钩不变。在确保绥棱摘帽和138个贫困村、70%以上贫困人口稳定脱贫的基础上，力争望奎、青冈、海伦摘帽，所有贫困村全部退出。

（七）加快社会事业发展，在改善保障民生上聚焦发力。突出抓好就业创业。城镇新增就业5万人，城镇登记失业率控制在4.3%以内。加大对零就业家庭和城镇就业困难人员的就业援助力度，做好离校未就业高校毕业生实名登记及就业服务工作。建立法律援助中心劳动人事争议仲裁工作站，构建和谐劳动关系。提高社会保障水平。支持符合条件的城乡居民参加养老保险。加快居民大病保险与医疗救助制度衔接。推行医养结合，鼓励社会资本建设大型护理院、老年病医院、康复医院等医养结合服务机构，新增养老床位2000张。加快宝山万康健康生态养老产业园建设。推进“最美慈善乡村”建设，争创“全国慈善城市”。加强安居工程建设。改造棚户区2.8万户、农村危房2万户。建设“选房超市”，提高货币化安置比例。通过新建、维修改造、购买或置换砖房、建设幸福大院等形式，兜底保障农村四类重点人群住房安全。鼓励农民进城购房，建立棚户区改造项目与普通商品房互通机制，消化存量商品房。加快各项事业发展。建设义务教育标准化学校30所，公办幼儿园10所，统筹发展职业教育。健全完善县、乡、村三级医疗卫生服务网络。实施文体惠民行动，大力推进体育产业发展，提高城乡居民健康素质。开展好雪地足球、滑冰比赛等系列“赏冰乐雪”活动。实施新一轮妇女儿童发展规划，进一步提高出生人口素质。

各位代表！为人民增福利、谋福祉是政府义不容辞的责任。我们将千方百计解决好广大群众生产生活中遇到的困难和问题。重点推进创业就业，让百姓有事做、有业就；抓好教育发展，让孩子读好书、能成才；加强医疗保障，让就医不难、看病不贵；改善人居条件，让群众住上暖屋子、有个好环境；完善社会保障，提高城乡低保、五保供养水平，让弱势群体有笑脸、有信心、有奔头。

市政府今年重点办好27件民生实事:①新建市内公共交通首末总站和充电站；②实施黄河路南段人行步道和绿化升级工程；③实施康庄路11公里沿线绿化升级改造工程；④完成高速路东出口绿化亮化工程；⑤新建府西绿地公园，实施6处街角绿化工程；⑥完成大唐热电联产项目建设，形成供热能力；⑦完成城区10公里供热管网建设，新增供热面积200万平方米；⑧改造棚户区20万平方米，购置公租房200套，使3016户居民和200户贫困家庭搬入新居；⑨完成10万平方米老旧小区宜居节能改造；⑩完成1.2万户居民城市管道天然气入户改造；⑪更新改造老城区12公里给水管网和15公里排水管网；⑫启动运营南城和东城客运站；⑬启动建设北郊污水处理厂；⑭续建环城绿化带，建设绿色屏障；⑮建设安装10处社区多功能健身场地设施；⑯完成第一中学电教馆建设；⑰新建1所幼儿园；⑱完成绥化肿瘤医院建设；⑲完成城市养老养护中心建设；⑳进一步提高低保补助标准，大幅提高高龄老人生活补贴标准；㉑开工建设中兴商业街地下停车场；㉒开工建设市第一医院儿科中心和第三住院部；㉓开工建设市人民办事中心；㉔实施老城区二次供水泵房改造工程；㉕打通科研路、黄河路，缓解市内交通压力；㉖启动市文化体育中心项目；㉗启动市博物馆改造工程。

这些民生实事都是老百姓期盼的事，也是对全市人民的郑重承诺，不管财力多么紧张，不管推进多么困难，我们都要坚决干，决不失信于人民！

（八）创新社会治理，在促进社会和谐稳定上聚焦发力。推进和谐社区建设。完善社区公共服务设施，改善社区办公条件，强化社区服务功能。推进民政、就业、社保、司法等便民机构进社区。开展以帮老、助残、环保、爱心等为主要内容的社区志愿者服务和社区自助、互助服务。加强社区图书室、文体活动场所、文化娱乐中心建设。每个县（市、区）打造2个以上精品社区，1个特色品牌社区。推进文明绥化建设。深入开展文明城市、文明村镇、文明行业、文明家庭、文明校园创建活动。开办道德讲堂、国学讲堂，倡导全民阅读，提高市民文化素养。强化诚信体系建设，加快建立个人信用体系。扶持文艺创作，推出一批反映绥化振兴发展的文艺精品。推进平安社会建设。用改革的办法健全矛盾纠纷化解机制，推进“法治信访”、“阳光信访”、“责任信访”，一手抓问题解决，一手抓秩序维护，认真解决群众合法诉求。强化安全生产基础保障能力和标准化建设，全面落实风险防控措施，突出整治重点领域、重点行业、重点部位安全隐患，坚决遏制重特大事故发生。加强公共安全、食品药品监管和质量监督。全面开展“七五”普法。构建立体化社会治安防控体系，严厉打击各类违法犯罪活动。建设平安绥化，确保城乡安全、群众安宁、社会安定。

四、加强政府自身建设

各位代表，面对新起点、新征程、新期待，我们将以更高的标准、更严的要求，坚持不懈加强政府自身建设，努力打造为民、务实、清廉、高效政府。

（一）加强能力建设，提高政府工作专业化水平。坚决避免少知而迷、无知而乱。以习近平总书记系列重要讲话精神，特别是对龙江两次重要讲话精神武装头脑，指导实践，推动工作。坚持问题导向，深刻理解党中央国务院、省委省政府出台的重大改革和政策措施，树牢市场理念和创新意识，准确把握新技术、新经济、新业态、新商业模式发展趋势，科学研判绥化优势潜力和国内国际竞争格局，增强工作的精准度、有效性，提高解决实际问题能力。大兴调查研究之风，注重在实践中提高本领。

（二）坚持依法行政，让政府工作在监督下阳光运行。切实增强法治、制度、规则意识，坚持运用法治思维、法治方式思考问题、解决问题。严格按照法定权限和程序行使权力。进一步健全公众参与、听证论证、风险评估机制，做到重大事项不论证不上会，重大民生工程不公开不实施，重大工程项目不评估不决策。自觉接受人大依法监督和政协民主监督，主动接受新闻舆论和社会公众监督，认真办理好代表委员建议提案。严格执行“两随机一公开”制度，规范行政执法自由裁量权，完善责任追究和责任倒查机制。加大政务公开力度，提高政府工作透明度。

（三）深入转变作风，努力提高政府行政效率。不断优化权力运行图和工作流程图，精减办事环节，压缩办结时限。简化涉建审批流程，建立民生领域事项简易程序，推进网上办公、网上审批，让数据多跑路，让企业和群众少跑腿。加强督办检查，专事专议，专题专责，重点解决久议不决、久拖不结。大力查处不作为、乱作为、慢作为，努力把绥化建设成为行政许可审批全省流程最优、时间最短、设限最少、效能最高的城市，真正使绥化的发展环境有一个大的改善，大的提升，树立良好新形象。

（四）敢于担当负责，提振干事创业精气神。强化正确目标导向，发扬钉钉子精神，把心思和精力凝聚到干事创业上来。树立功成不必在我的理念，多做打基础、利长远的工作。建立完善正向激励机制，促进干部想干事、敢干事，让履职尽责、干事创业蔚然成风。坚持问政于民、问需于民、问计于民，调动激发方方面面的智慧力量，大家撸起袖子加油干。

（五）切实干净干事，建设廉洁政府。坚定政治方向，强化“四个意识”，坚决拥护以习近平同志为核心的党中央。严格落实“一岗双责”，加强政府系统党风廉政建设。严格管控廉政风险，严格项目评审，把财政资金花好，花出最大效用。建好公共资源交易中心，建立与市场经济相适应的交易机制，公开规范项目招投标、土地出让、政府采购等公共资源交易。加强重点领域、关键环节的行政监察和审计监督，严肃查处顶风违纪行为。维护山清水秀的政治生态。

各位代表！今天的绥化，已经踏上转型升级、振兴发展的新征程。浪开行远，扬帆起航。让我们在省委、省政府和市委的坚强领导下，凝心聚力、砥砺前行、决战决胜，奋力谱写绥化全面振兴发展新篇章！
